# Radiofrequency electromagnetic field ınhibits HIF-1 alpha and activates eNOS signaling to prevent intestinal damage in a model of mesenteric artery ischemia in rats

**DOI:** 10.7150/ijms.105479

**Published:** 2025-02-26

**Authors:** Eyyup Sabri Ozden, Mustafa Soner Ozcan, Ilter Ilhan, Muhammet Yusuf Tepebasi, Rumeysa Taner, Dincer Uysal, Halil Asci, Selcuk Comlekci, Ozlem Ozmen

**Affiliations:** 1 Department of Anesthesiology and Reanimation, Faculty of Medicine, Suleyman Demirel University, Isparta, Turkey.; 2 Department of Medical Biochemistry, Faculty of Medicine, Suleyman Demirel University, Isparta, Turkey.; 3 Department of Medical Genetics, Faculty of Medicine, Suleyman Demirel University, Isparta, Turkey.; 4 Department of Bioengineering, Institute of Science and Technology, Suleyman Demirel University Isparta, Turkey.; 5 Department of Cardiovascular Surgery, Faculty of Medicine, Suleyman Demirel University, Isparta, Turkey.; 6 Department of Pharmacology, Faculty of Medicine, Suleyman Demirel University, Isparta, Turkey.; 7 Department of Pathology, Faculty of Veterinary Medicine, Burdur Mehmet Akif Ersoy University, Burdur 15030, Turkey.

**Keywords:** Apoptosis, Electromagnetic field, Mesenteric ischemia, Oxidative stress Radiofrequency

## Abstract

**Background:** Pathologies such as mesenteric artery ischemia and reperfusion (MIR) can lead to many organ dysfunctions, including the brain and heart through damage mechanisms induced in response to hypoxic conditions. Radiofrequency electromagnetic field (RF-EMF) can increase the vascularization of tissues by providing endothelial nitric oxide synthase (eNOS)-mediated nitric oxide (NO) release from the endothelium. The aim of this study is to investigate the protective effect and mechanism of RF-EMF in ischemic intestinal injury in the experimental MIR model.

**Methods**: In the study, 32 Wistar Albino rats were divided into four groups: Sham group, MIR group, Prophylactic (Pr) RF-EMF + MIR group, MIR + Therapeutic (Tr) RF-EMF group. At the end of the experimental phase, after sacrifice, blood samples and the 10 cm terminal ileum part of the intestinal tissues was cut and collected for histopathological, immunohistochemical, genetic and biochemical analyses.

**Results**: In the MIR group, Cas-3, TNF-α, VEGF, BAX and HIF-1α expressions increased, while OSI levels, and PCNA, BCL2 and eNOS expressions decreased. In addition marked hyperemia, hemorrhage, edema, inflammatory cell infiltrations, and erosion or ulcers were observed in MIR group. Pr (especially in eNOS expression) and Tr (especially in pathological findings) treatment of RF-EMF reversed all these parameters but more effective recovery was observed in Tr treated group.

**Conclusion:** RF-EMF-treatment preserved the vascularization of the tissue and decreased hypoxia-induced oxidative stress, inflammation, and apoptosis.

## Introduction

It is known that all substances taken orally are absorbed at the level of the small intestine and then enter the systemic circulation via the portal vein first and then via the inferior vena cava 1. In cases of damage that may occur in the intestinal tissue, which has such an important localization, it is possible that the liver and distant organs via the blood will be affected by this damage due to the mentioned route. Moreover, damages in many tissues including the brain and heart caused by pathologies such as ischemia and reperfusion of the mesenteric artery (MIR) in the intestine may cause dysfunctions in these organs 2. It is known that in such damages occurring in the villi structures in the intestinal tissue, drugs taken orally for treatment cause rapid passage through the intestinal passage due to increased peristaltic movements and decreases in drug efficacy 3.

Apart from MIR, the etiology of this event includes splanchnic vasoconstriction, non-occlusive mesenteric disease, mesenteric thrombosis and embolic events in mesenteric vessels from chronic arterial occlusion 4. Depending on the extent of ischemia, anastomotic leaks may also cause death by affecting postoperative mortality in the entire gastrointestinal system 5.

In the treatments applied in MIR, thrombus causing ischemia is formed with platelet aggregation occurring after plaque rupture, substances such as serotonin, thromboxane A2, and thrombin released from these platelets and endothelial nitric oxide synthase (eNOS)-mediated nitric oxide (NO)-mediated vasoconstriction in the lesion area and ischemia-induced oxygenation of the distal tissue may be further impaired 6,7.

Intracellular pathways activated by increased free oxygen radicals-induced oxidative stress and complement system-induced inflammation are responsible for the oxidant and inflammatory response occurring in the distal intestinal tissue due to hypoxia. The continuity of hypoxic conditions occurring in the distal tissue due to ischemia may trigger intracellular pathways such as hypoxic-induced factor-1 alpha (HIF-1α) and lead to the expression of some markers such as tumor necrosis factor-alpha (TNF-α), interleukin-1 (IL-1) and interleukin-6 (IL-6) that may trigger inflammation 8,9.

On the other hand, it is known that damage to the mitochondria, which have important roles in oxidative phosphorylation and ATP production at the cellular level, leads to an increase in BCL2 Associated X (BAX) gene expression and a decrease in B-cell lymphoma 2 (BCL2) gene expression in the cell membrane and apoptosis occurs as a result of increased mitochondrial membrane permeability. As stated in many publications in the literature, these damage mechanisms can trigger each other by activating intracellular pathways and cause the damage to show a cumulative and progressive course 10-12. Caspase-3 is a frequently activated death protease, catalyzing the specific cleavage of many key cellular proteins. Pathways to caspase-3 activation have been identified that are either dependent on or independent of mitochondrial cytochrome c release and caspase-9 function. Caspase-3 is essential for normal brain development and is important or essential in other apoptotic scenarios in a remarkable tissue-, cell type- or death stimulus-specific manner. Caspase-3 is alsorequired for some typical hallmarks of apoptosis, and is indispensable for apoptotic chromatin condensation and DNA fragmentation in all cell types examined. Thus, caspase-3 is essential for certain processes associated with the dismantlingof the cell andthe formation of apoptotic bodies, but it may also function before or at the stage when commitment to loss of cell viability is made 13.

**Figure 1:**
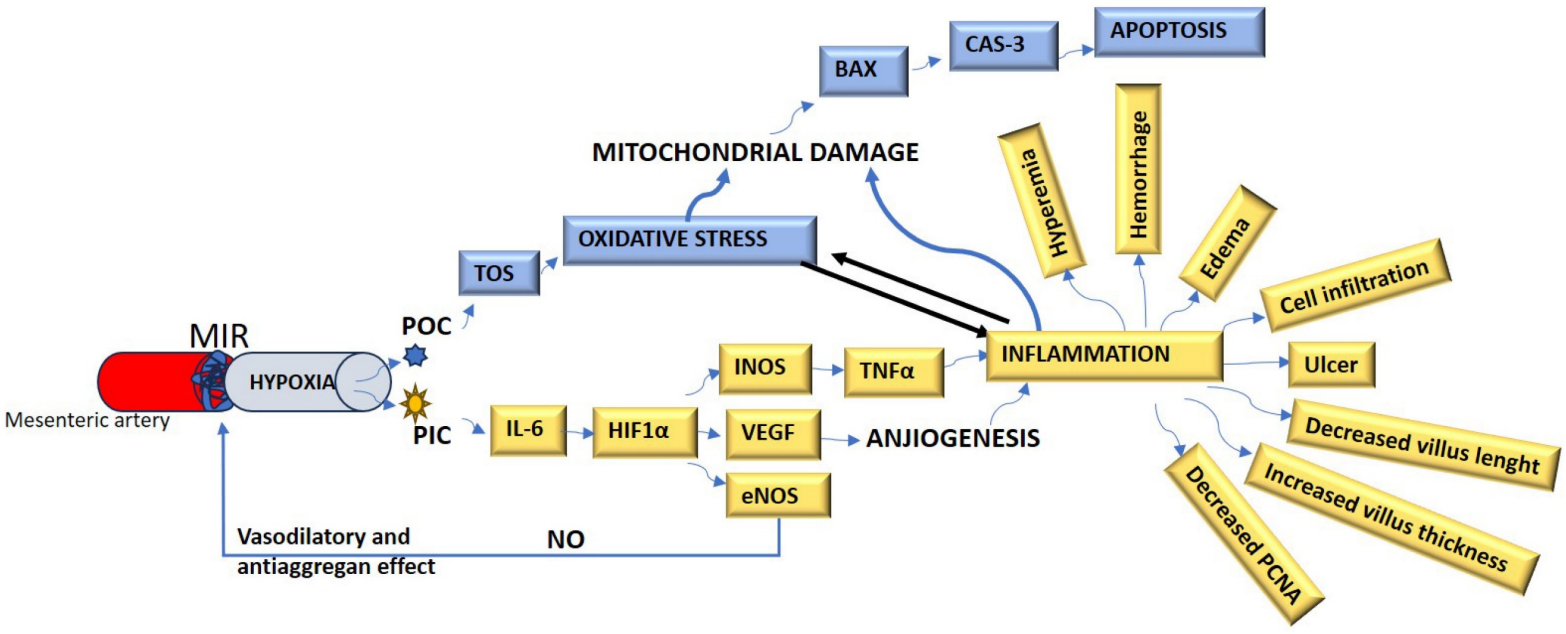
The summarize of the cell damage mechanism in MIR. *MIR: Mesenteric artery ischemia and reperfusion, POC: Prooxidant cytokines, PIC: Proinflammatory cytokines, TOS: Total oxidant status, IL-6: Interleukin-6, NO: Nitric oxide, HIF-1α: Hypoxic-induced factor-1 alpha, INOS: Inducible nitric oxide synthase, VEGF: Vascular endothelial growth factor, eNOS: endothelial nitric oxide synthase, BAX: B-cell lymphoma 2 Associated X, TNF-α: Tumor necrosis factor-alpha, Cas-3: Caspase-3, PCNA: Proliferating cell nuclear antigen*.

The connections of all the biomarkers mentioned above with each other and with cell damage mechanisms are summarized in Figure 1.

Shortwave Diathermy (SWD), or more commonly referred to as "shortwave," is one of the modalities used in physical therapy. Diathermy is a general term for physical therapy methods aimed at achieving therapeutic effects in various muscle, joint, and soft tissue disorders by heating deep body tissues. It generates heat through high-frequency electromagnetic energy, utilizing currents with frequencies of 27.12 MHz and a wavelength of 11.06 meters, or 22 MHz and a wavelength of 7.5 meters. The application can be in the form of continuous or pulsed energy delivery. Continuous energy flow produces greater heating effects, while pulsed energy can exhibit mechanical effects independent of thermal influence. Non-thermal effects of pulsed currents include the acceleration of cell growth, enhancement of wound healing, and repair of damaged cells. SWD therapy may be utilized in the physical treatment of conditions such as muscle spasms, strains, bursitis, tenosynovitis, arthritis, lower back pain, and fibromyalgia.

Radiofrequency (RF) has been recognized for achieving favorable clinical outcomes in dermatology as a surgical and/or ablation method. In a study, the clinical efficacy and safety of 27.12 MHz RF in the treatment of benign cutaneous lesions were evaluated by including twenty female patients. Clinical outcomes were assessed after a single session of treatment with 27.12 MHz RF, as well as at 1 and 3 weeks post-treatment. The treated lesions included telangiectasia, port-wine stain, skin tags, seborrheic keratosis, lentigo, milia, enlarged pores, acne, piercing holes, and a case of neurofibroma. The treatment of benign vascular and non-vascular lesions with 27.12 MHz RF appeared to be safe and effective following a 3-week follow-up period 14.

Physiological and anatomical differences between rats and humans can influence the interaction of electromagnetic waves with biological tissues. Factors such as the diameter of mesenteric vessels, the electrical properties of tissues, and the resonance frequency are particularly critical in determining electromagnetic energy absorption (Specific Absorption Rate, SAR). Experimental studies must first be conducted to verify whether a frequency identified in humans induces the same biological effects in rats.

The radiofrequency electromagnetic field (RF-EMF) at a frequency of 27.12 MHz, the effect of which was examined in this study and which is routinely approved by the FDA and included in some devices such as short-wave diathermy, has been proven to cause an increase in NO synthesis and vasodilation in the vessels by increasing the expression of eNOS enzyme in the endothelial layer, the innermost layer of vascular structures in the area where it is applied 15. It also shows its vasodilator effect by increasing the exit of calcium from the cell and its entry into the sarcoplasmic reticulum 16. On the other hand, it contributes to angiogenesis by increasing HIF-1α-mediated vascular endothelial growth factor (VEGF) levels in addition to its NO-mediated effect 15. NO produced from L-arginine activates guanylate cyclase and increases cGMP levels, resulting in protein kinase G activation and phosphodiesterase inhibition and decreases platelet aggregation and adhesion 17.

The aim of this study was to investigate the effect of prophylactic (Pr) and therapeutic (Tr) RF-EMF application, a noninvasive, sensitive and specific method that can increase NO production, in an experimental mesenteric ischemia model. In addition, mitochondrial involvement and HIF-1α signaling, which are potential mechanisms for this effect, were investigated.

## Materials and Methods

### Ethical approval

The procedures performed on rats were reviewed and approved by the Animal Experiments Local Ethics Committee of Suleyman Demirel University (Ethic No: 11.05.2023-05/169). The experiment was conducted according to the ARRIVE (Animal Research: Reporting *In Vivo* Experiments) guidelines, Version 2.0 protocol. In addition, Suleyman Demirel University Scientific Research Project Unit supported the study with the project number TSG-2023-9092.

### Animals

Thirty-two adults female Wistar Albino rats weighing 300-350 g were housed in standard Euro-type 4 cages, each group separated from the other. They were housed at 23 °C and 55% humidity with 12 hours light and 12 hours dark cycle. They were fed ad libitum with a standard commercial feed and water. The four experimental groups were formed as follows:

*1-Sham group (n=8):* RF-EMF unit was inactivated. After 30 min, the abdominal regions of the animals were opened with abdominal incision under anesthesia and ischemia model was not applied. After 150 min (30 min + 120 min), the animals were sacrificed under anesthesia and intestinal tissues were removed.

*2- Mesenteric Ischemia and Reperfusion (MIR) group (n=8):* RF-EMF unit was inactivated. After 30 minutes, the animals were anesthetized, the abdominal skin of the rats was carefully shaved and cleaned before the operation. After preparing the skin with povidone-iodine, a 5 cm midline incision was made. In the groups undergoing MIR, the abdominal walls were manipulated with 4-0 silk sutures at intervals. In other groups, the superior MIR was identified and dissected. Following MIR dissection, an atraumatic microvascular clamp was placed along the origin of the MIR divergence from the abdominal aorta. MIR was confirmed by the absence of arterial pulse and intestinal pallor. The animals were left to reperfusion at 30 min after ischemia and after 120 min, the animals were sacrificed under anesthesia and intestinal tissues were removed 18.

*3- Prophylactic RF-EMF + MIR group (n=8):* After 30 minutes of active RF-EMF application, rats were subjected to ischemia with the help of bulldog clamp under anesthesia. The animals were left to reperfusion at 30 min after ischemia and after 120 min, the animals were sacrificed under anesthesia and intestinal tissues were removed 15,18.

**Figure 2:**
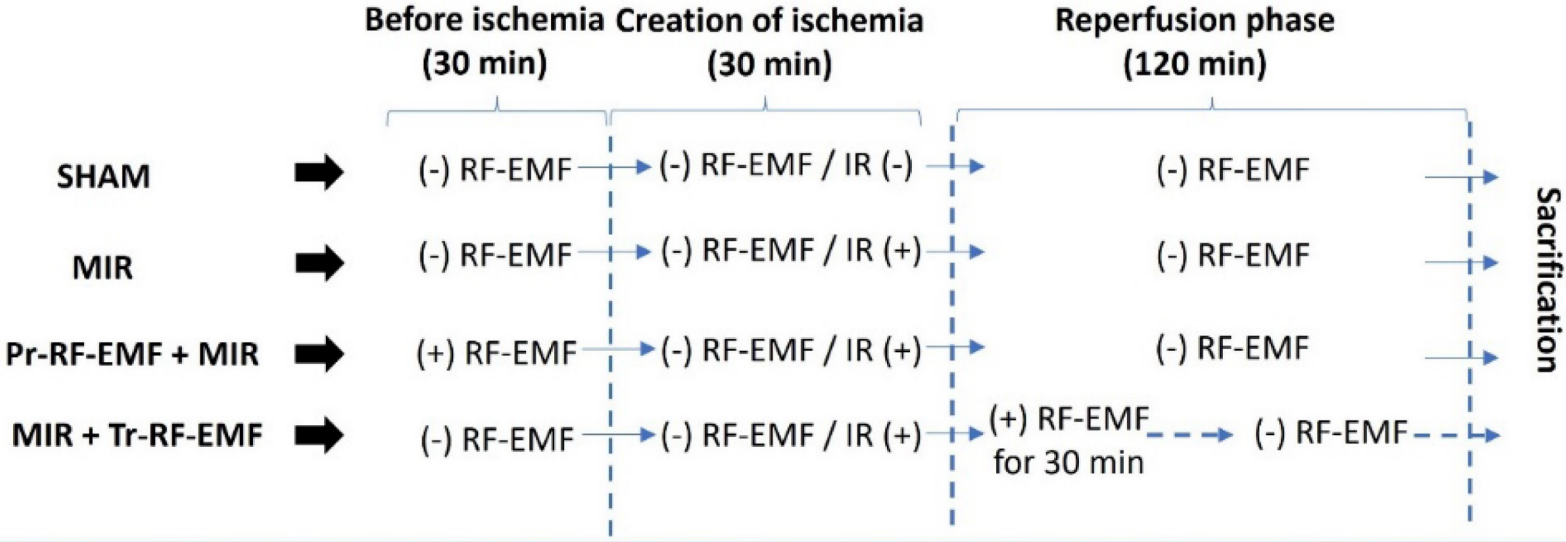
Experimental procedures of the study. *MIR: Mesenteric Ischemia and Reperfusion group, Pr-RF-EMF: Prophylactic radiofrequency electromagnetic field group, Tr-RF-EMF: Therapeutic radiofrequency electromagnetic field group*.

* 4- MIR + Therapeutic RF-EMF group (n=8):* The animals were kept in the inactive RF-EMF unit for 30 min and ischemia was applied with the help of bulldog clamp under anesthesia. After the RF-EMF application, which was activated at the 30th minute after ischemia, was applied for 30 minutes, the animals were kept for 90 minutes, sacrificed and intestinal tissues were removed (Figure 2).

At the sacrifice stage, following the abdominal incision under 90 mg/kg Ketamine (Keta-Control, Doğa İlaç, Turkey) and 8-10 mg/kg Xylazine (Xylazin Bio 2%, Bioveta, Czech Republic) anesthesia, surgical exsanguination performed by taking blood from the vena cava inferior. The 10 cm terminal ileum of the intestinal tissues were cut and removed. Half of the tissues were put into 10% formaldehyde for histopathological analyses as hematoxylin eosin (HE) staining and immunohistochemical evaluations for TNF-α, proliferating cell nuclear antigen (PCNA) and caspase-3 (Cas-3) expressions. The remaining tissues were put into -80℃ for biochemical analyses as total oxidant status (TOS), total antioxidant status (TAS) and oxidative stress index (OSI), genetic analyses as eNOS, HIF-1α, VEGF, BCL2 and BAX gene expressions.

### RF-EMF setup and applications

Radiofrequency field exposure (RF-EMF) was planned as 27.12 MHz energy. We used a specially made device to generate RF-EMF electromagnetic energy level. This device is a special generator that can generate a very homogeneous 27.12 MHz frequency and 0.8 power output. The exposure lasted only one day and 30 minutes. The homogeneous magnetic field was suitable for the dimensions of the Euro type-4 cage and 30 mm above the cage bottom using electromagnetic excitations (CST Studio Suite 3D simulation, Oxford shire, England). A 27.12 MHz RF applicator was used to obtain an electric field value of 10 V/m. In this case, we obtained 0.8 W RF energy from the special PCB antenna. RF-EMF measurements were performed using a field meter (ELF meter PF-4, England). Spectrum analyzers (ROHDE-SCHWARZ FSH6, USA, PROMAX AE-566 spectrum analyzer, USA) were used in each stage of the experimental steps. All experiments were carried out in an electromagnetic isolated room. This type of shielding room is located in the animal experiments laboratory of the medical faculty. This shielding room is suitable for this type of electromagnetic studies. The shielding effectiveness value of this experimental room was measured as 80 dB at the operating frequency of 27.12 MHz.

### Biochemical analysis

Intestine tissue samples were homogenized with Ultra Turrax Janke&Kunkel T-25 homogenizer (IKA®-Werke, Germany) for oxidant-antioxidant analysis. TAS, TOS were measured spectrophotometrically (Beckman Coulter AU 5800, Beckman Coulter, USA) by using commercial kits (Rel Assay Diagnostics, Gaziantep, Turkey) and OSI was calculated using the formula OSI = TOS/TAS 19,20. For TAS analysis, antioxidants in the sample reduce dark blue-green colored 2,2'-azino-bis (3-ethylbenzthiazoline-6-sulphonic acid) (ABTS) radical to colorless reduced ABTS form. The change of absorbance at 660 nm is related to the total antioxidant level of the sample. This method determines the antioxidative effect of the sample against the potent free radical reactions initiated by the produced hydroxyl radicals. The results are expressed as millimolar Trolox equivalent per liter. For TOS analysis, oxidants present in the sample oxidize the ferrous ion- dianisidine complex to the ferric ion. The oxidation reactions are enhanced by glycerol molecules, which are abundantly present in the reaction medium. The ferric ion forms a colored complex with xylenol orange in an acidic medium. The color intensity, which could be measured spectrophotometrically, has been found related to the total amount of oxidant molecules which were already present in the sample. The assay was calibrated with hydrogen peroxide, and the results were expressed in terms of micromolar hydrogen peroxide equivalent per liter (µmol H_2_O_2_ Eqv/L) 21.

### Histopathological evaluations

At the conclusion of the investigation, samples of small intestine tissue were collected for histological analysis. These tissues were fixed in 10% buffered formalin and processed using automatic tissue processing equipment (Leica ASP300S; Leica Microsystems, Nussloch, Germany). Following paraffin embedding, the samples were sectioned at 5 µm using a Leica RM 2155 rotary microtome (Leica Microsystems, Nussloch, Germany). The sections were stained with hematoxylin and eosin (HE) and then examined under a microscope.

For morphometric examination, ten randomly chosen villi from each rat's intestines were selected. The length and thickness of the villus, were measured at 40x magnification using an Olympus CX21 light microscope. The average of these scores was considered the score for each rat. The morphometric evaluation was performed using the Database Manual Cell Sens Life Science Imaging Software System (Olympus Corporation, Tokyo, Japan).

Each rat intestinal segment was scored based on semiquantitative measurements of hyperemia, hemorrhage, edema, inflammatory infiltration, and epithelial injury. The scoring system ranged from 0 to 3, based on severity. The criteria for scoring are presented in Table 1 22.

### Immunohistochemical analysis

The streptavidin-biotin complex peroxidase method was used to stain three separate serial sections of intestinal tissues, which were placed on Poly-L-lysine slides. Primary and secondary antibodies were provided by Abcam (Cambridge, UK). Immunohistochemical staining was performed according to the manufacturer's instructions to determine the expression of Caspase-3 ([Recombinant Anti-Caspase-3 p12 antibody [EPR16888] (ab179517)], PCNA (Anti-PCNA antibody [PC10], ab29], and TNF-α (Anti-TNF alpha antibody [EPR21753-109, ab205587], 1/100 dilution). The EXPOSE Mouse and Rabbit Specific HRP/DAB Detection IHC kit (ab80436) from Abcam was used as the secondary antibody. Additionally, the chromogen diaminobenzidine (DAB) was employed. For the negative controls, an antigen dilution solution was used instead of the primary antibody. After calculating the average proportion of cells stained by each antibody, the groups were compared. For each rat, 100 cells were counted at a 20X magnification in five randomly selected gut regions (20 cells each). The only cells considered affirmative were those that had a noticeable brown stain. Each positive cell in the target area was individually clicked for counting, and the software then marked and counted each cell. To count positive cells in the intestine, Image J 1.48 software (National Institutes of Health, Bethesda, MD) was utilized.

### Reverse transcription-polymerase chain reaction (RT-qPCR)

Using the GeneAll RiboEx (TM) RNA Isolation Kit (Cat No: 301-001) and the manufacturer's instructions, RNA was extracted from homogenized tissues (GeneAll Biotechnology, Seoul, Korea). A BioSpec-nano NanoDrop UV-Vis spectrophotometer (UV-2600, Shimadzu Ltd. Kyoto, Japan) instrument was used to measure the quantity and purity of the RNAs that were collected. cDNA synthesis was performed using the A.B.T.™ cDNA Synthesis Kit (Cat No: C03-01-05) from Atlas Biotechnology in Turkey according to the instructions. One microgram of RNA was used for cDNA synthesis. Using the Primer-BLAST tool, NCBI website, specific mRNA sequences were found, and potential primer sequences were then tested. In Table 2, the primers' sequences used, accession numbers, and product size of the genes are given. A.B.T.™ SYBR Master Mix (Atlas Biotechnology, Turkey) (Cat No: Q04-01-05) was used to quantify the expression levels of genes in a Biorad CFX96 real-time PCR equipment (CA, USA). In the study, the glyceraldehyde-3-phosphate dehydrogenase (GAPDH) gene was used as a housekeeping gene. The reaction mixture was prepared according to the manufacturer's protocol to a final volume of 20 µL. The resultant reaction mixture was put into a real-time qPCR equipment with thermal cycling setup in accordance with the manufacturer's protocol for the kit, and each sample was examined in three replications. The PCR protocol was applied as 1 cycle with an initial denaturation at 95 °C for 300 s and 40 cycles with denaturation at 95 °C for 15 s, annealing/extension at 55 °C for 30 s. Relative mRNA levels were calculated by applying the 2-^ΔΔ^Ct formula to the normalize the results.

### Statistical analysis

To determine if the data distribution was normally distributed, the Shapiro-Wilk method was first used. The data displayed a normal distribution (P>0.05), hence an ANOVA was used to compare the groups. One-way ANOVA post hoc Tukey test using the Graphpad Prism program was utilized for the group comparison statistical analysis, and p<0.05 was deemed significant.

## Results

### Biochemical results

**Figure 3:**
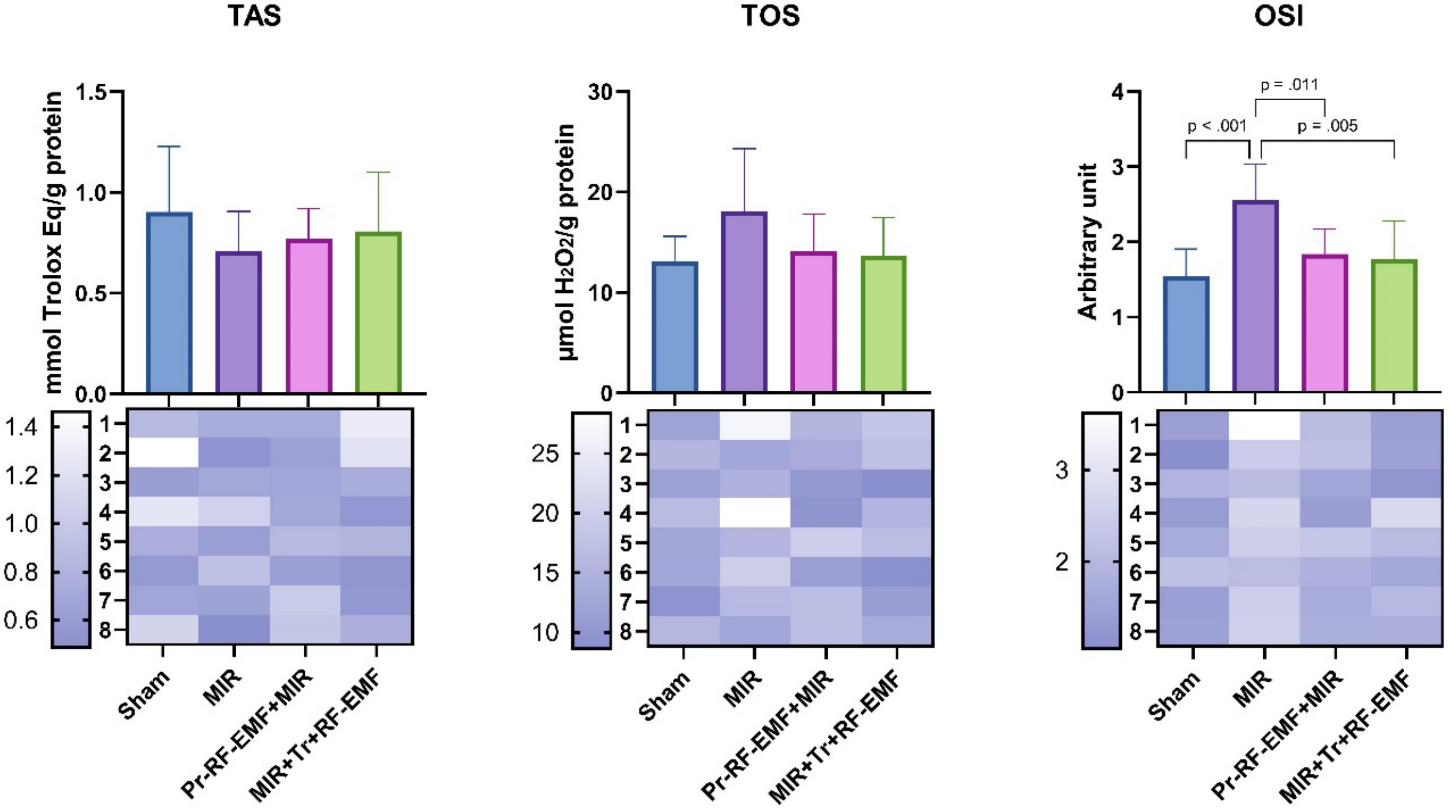
Oxidative stress parameters of the study. *MIR: Mesenteric Ischemia and Reperfusion group, Pr-RF-EMF: Prophylactic radiofrequency electromagnetic field group, Tr-RF-EMF MIR: Therapeutic radiofrequency electromagnetic field group, TAS: Total antioxidant status, TOS: Total oxidant status, OSI: Oxidative stress index, Values are presented as means ± standard deviation. Oneway ANOVA test was used*.

When TOS levels, one of the indicators of oxidative stress, were analyzed, there was no significant difference between the groups. TAS values, which are indicators of antioxidant activity, showed no significant difference between the groups. For OSI values, a significant increase was found in the MIR group compared to the sham group (p<0.001). There was no significant difference between the treatment groups and the sham group. In addition, Pr and Tr application of RF-EMF significantly decreased OSI levels compared to MIR group (p=0.011 and p=0.005) (Figure 3).

### Histopathological results

**Figure 4:**
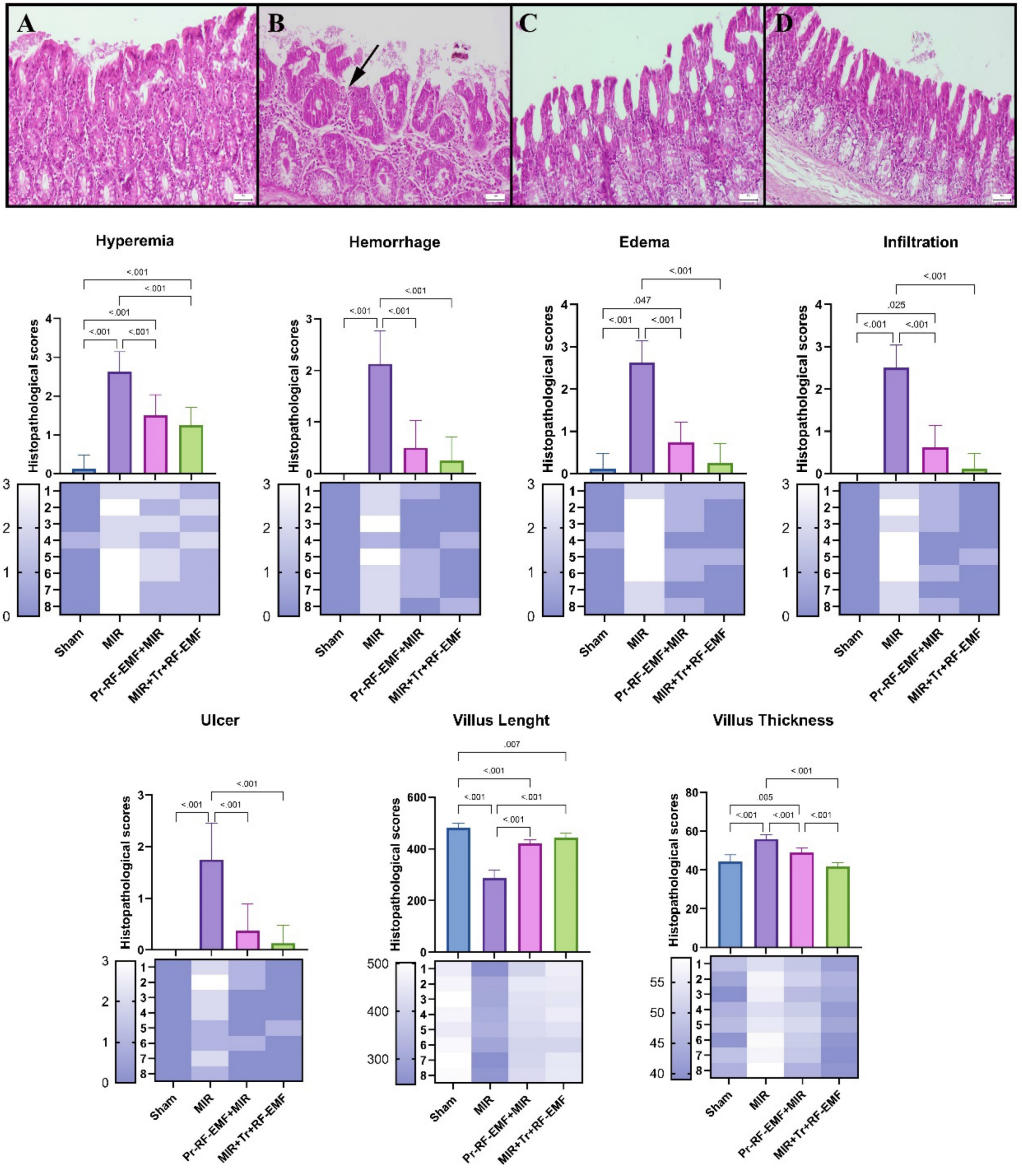
Representative histopathological view of the intestine*. (A) Normal histology in the control group. (B) Marked ulcers, edema, and inflammatory cell infiltrations (arrow) in the MIR group. (C) Significant decrease in pathological findings in the Pr-RF-EMF group. (D) Almost normal appearance in the Tr-RF-EMF group. HE, Scale bars = 50 μm. MIR: Mesenteric Ischemia and Reperfusion, Pr-RF-EMF: Prophylactic radiofrequency electromagnetic field, Tr-RF-EMF: Therapeutic radiofrequency electromagnetic field.*

Histopathological examination of the intestines revealed a normal microscopic appearance in the sham group. In the MIR group, marked hyperemia, hemorrhage, edema, inflammatory cell infiltrations, and erosion or ulcers were observed compared to sham group (p<0.05 for all). In severe ulcer areas, villus length was reduced due to villus shedding and villus thickness generally increased in lesion areas due to hyperemia, edema, and infiltrations in MIR group compared to sham group (p<0.001 for both). The Tr treatment of RF-EMF was found to be more effective than Pr treatment of RF-EMF alone compared to MIR group (Figure 4).

### Immunohistochemical results

**Figure 5:**
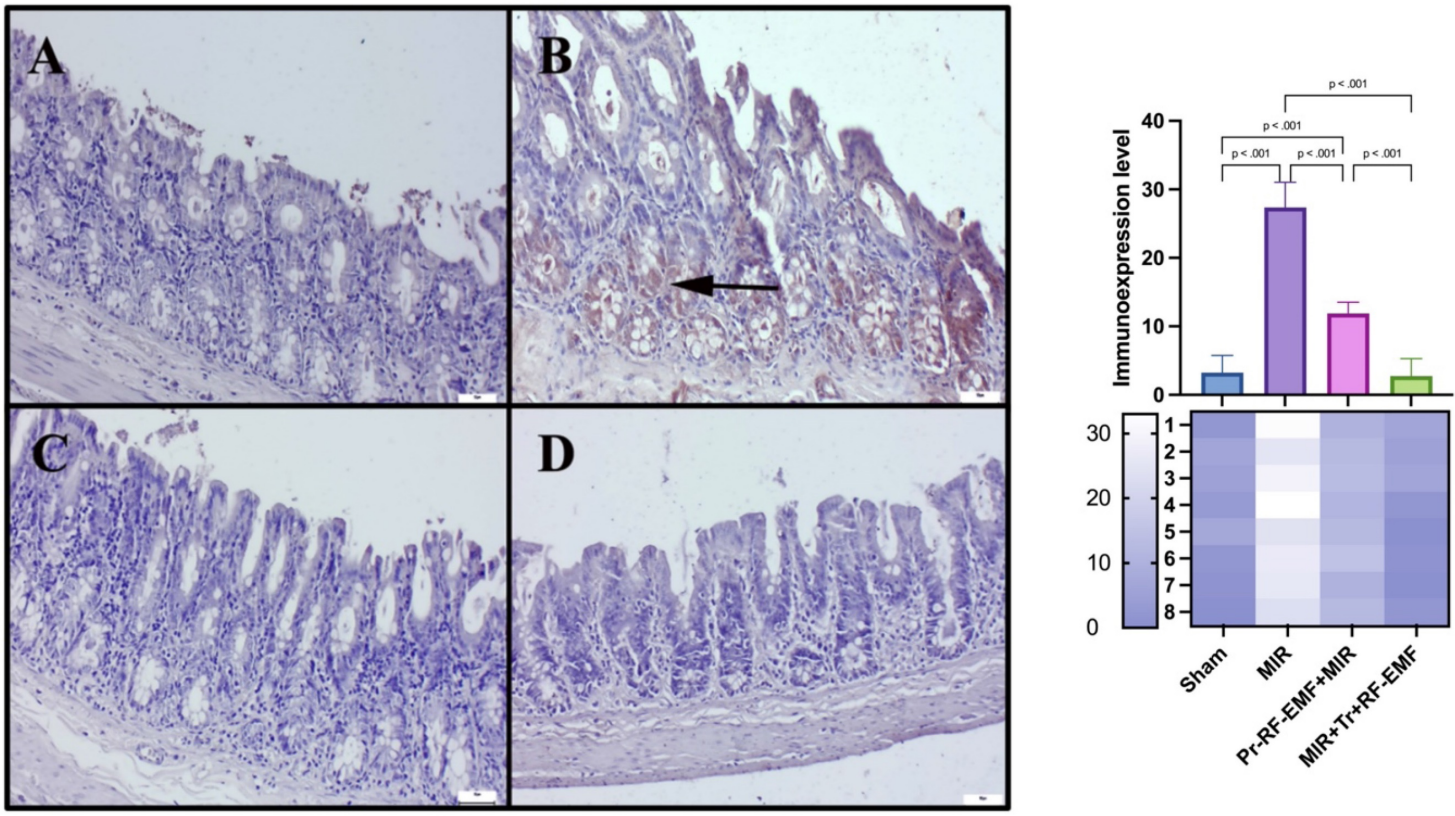
Cas-3 expressions among the groups. *(A) Very slight Cas-3 expressions in the sham group. (B) Marked increase in Cas-3 in the MIR group. (C) Slight Cas-3 in the prophylactic RF group. (D) Negative Cas-3 expressions in the therapeutic RF group. Scale bars = 50 μm. MIR: Mesenteric Ischemia and Reperfusion, Pr-RF-EMF: Prophylactic radiofrequency electromagnetic field, Tr-RF-EMF: Therapeutic radiofrequency electromagnetic field*.

**Figure 6:**
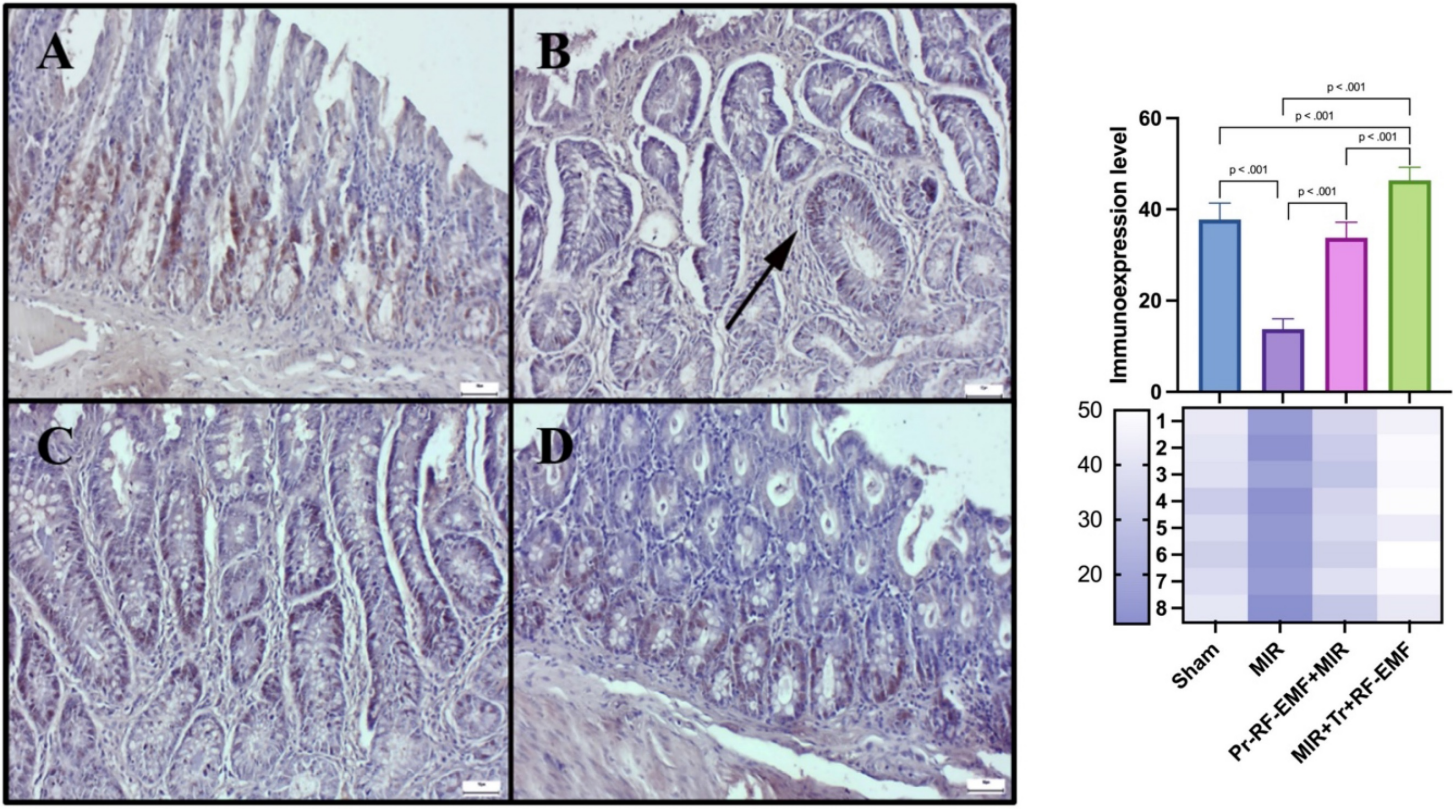
PCNA expressions among the groups. *(A) Very slight PCNA expressions in the sham group. (B) Marked decrease in PCNA expressions in the MIR group. (C) Slight PCNA expressions in the prophylactic RF group. (D) PCNA expressions in the therapeutic RF group. Scale bars = 50 μm. MIR: Mesenteric Ischemia and Reperfusion, Pr-RF-EMF: Prophylactic radiofrequency electromagnetic field, Tr-RF-EMF: Therapeutic radiofrequency electromagnetic field*.

**Figure 7:**
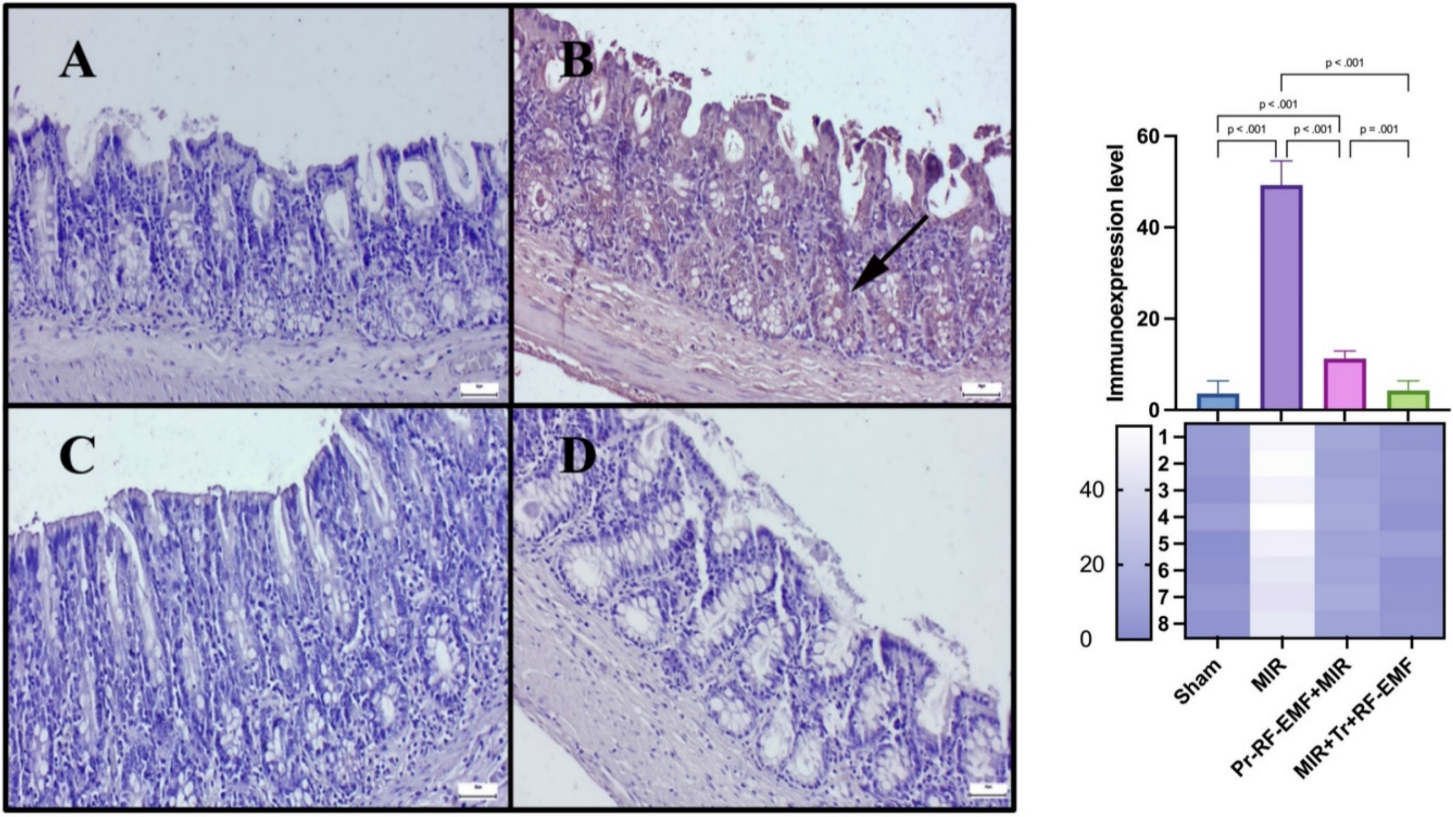
TNF-α expressions among the groups. *(A) Very slight TNF-α expressions in the sham group. (B) Marked increase TNF-α expressions in the MIR group. (C) Slight TNF-α expressions in the prophylactic RF group. (D) Negative TNF-α expressions in the therapeutic RF group. Scale bars = 50 μm. MIR: Mesenteric Ischemia and Reperfusion, Pr-RF-EMF: Prophylactic radiofrequency electromagnetic field, Tr-RF-EMF: Therapeutic radiofrequency electromagnetic field*.

Immunohistochemical analysis revealed slight to negative Cas-3 and TNF-α expressions, but marked PCNA expressions in the sham group. MIR caused a marked increase in Cas-3 and TNF-α expressions and a decrease in PCNA expressions compared to sham group (p<0.001 for all). Pr-RF-EMF caused a decrease in Cas-3 and TNF-α expressions and an increase in PCNA expressions compared to sham group (p<0.001 for all). The Tr-RF-EMF treatment resulted in a more pronounced improvement in expressions compared to sham group (p<0.001 for all). Cas-3 and TNF-α expressions were observed to be intracytoplasmic, while PCNA expressions were localized intranuclearly (Figure 5-7).

### Genetic results

**Figure 8:**
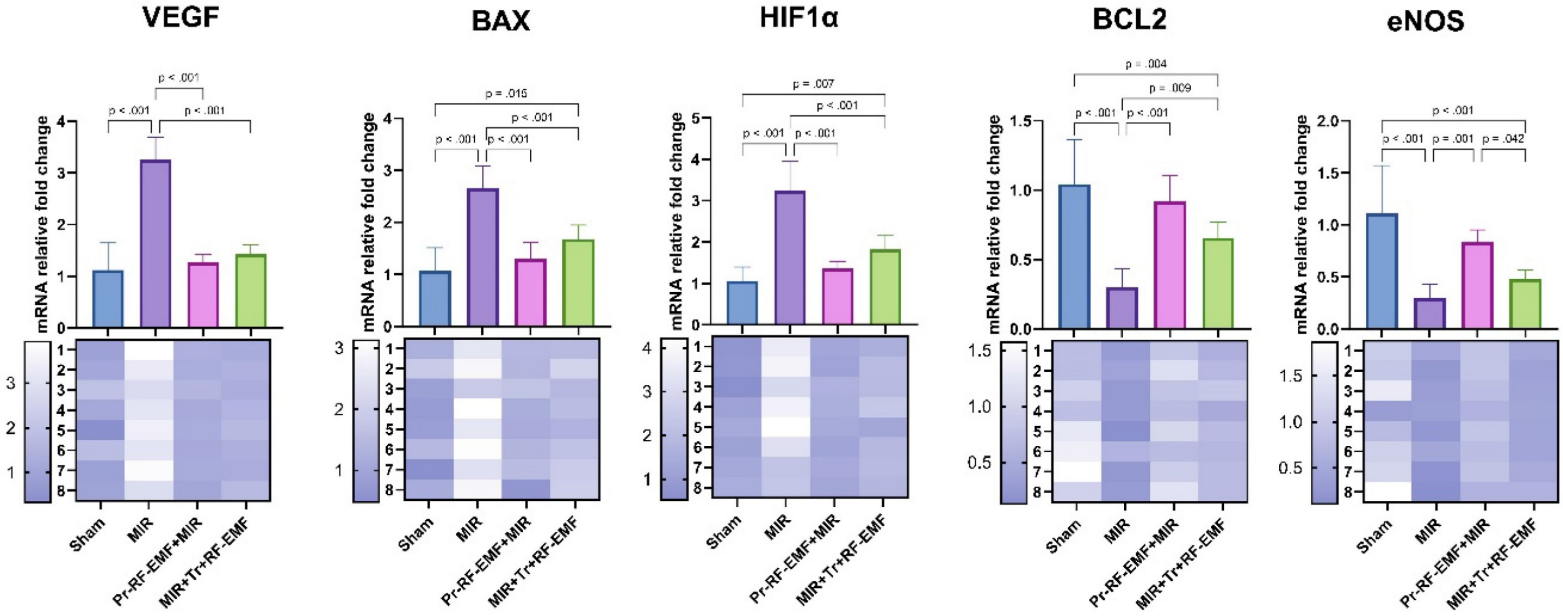
mRNA expression levels of tissues. *MIR: Mesenteric Ischemia and Reperfusion group, Pr-RF-EMF: Prophylactic radiofrequency electromagnetic field, Tr-RF-EMF Therapeutic radiofrequency electromagnetic field, BAX: BCL2 Associated X, BCL2: B-cell lymphoma 2, HIF1 α: Hypoxia-inducible factor 1 alpha, VEGF: Vascular Endothelial Growth Factor, eNOS: Endothelial nitric oxide synthase*.

Genetic analysis showed a statistically significant increase in VEGF, BAX, HIF1α gene expressions in the MIR group compared to the sham group, while a significant decrease was found in BCL2 and eNOS gene expressions (p<0.001 for all). On the other hand, VEGF, BAX, HIF1α gene expressions showed a statistically significant decrease in Pr-RF-EMF and Tr-RF-EMF groups compared to MIR group (p<0.001 for all). While an increase was observed in BCL2 and eNOS gene expressions in the Pr-RF-EMF group compared to the MIR group (p<0.001 for both), only BCL2 expression was significant in the Tr-RF-EMF group compared to the MIR group (p=0.009). In addition, BAX and HIF1α expressions increased in the MIR+Tr+RF-EMF group compared to the sham group (p=0.015; p=0.007), while BCL2 and eNOS expressions decreased (p=0.004; p<0.001). When the expression differences between treatment groups were analyzed, the only significant difference was an increase in eNOS expression in Pr-RF-EMF+MIR group compared to MIR+Tr+RF-EMF group (p=0.042) (Figure 8).

## Discussion

Mesenteric artery ischemia can be caused by a variety of coagulative problems and may lead to clinical conditions ranging from simple dysfunction of the intestinal tissue to death. Anticoagulant therapies may also be used to dissolve the clot formed in treatment modalities that may end with bowel resections 23.

Oxidant and inflammatory reactions triggered by the hypoxic environment in the intestinal tissue may cause hyperemia, hemorrhage and edematous pictures secondary to inflammatory cell migration and increased thickness of the villus structures forming the absorption surface. Ulcerative reactions triggered by apoptotic and necrotic reactions may also cause disruption of the absorption surface and shortening of villus lengths 24. Considering the histopathologic data of this study, hyperemia and hemorrhage foci detected in the MIR group provide important information about the depth of damage. It can be mentioned that intense inflammation with inflammatory cell infiltration and increased capillary membrane permeability caused by cytokines lead to edema. Increased villus thickness due to the same infiltration and edema may be one of the main causes of possible functional loss of intestinal tissue. Immunohistochemical analysis revealed that the expression of inflammatory markers and acute phase protein TNF-α were significantly increased in the MIR group compared to the sham group, supporting the histopathologic findings.

On the other hand, it is known that inflammation and oxidative stress can also stimulate apoptosis, which is a more dangerous picture 25. Cas-3 increases in the MIR group detected in immunostaining indicate the development of apoptosis in the tissue. It seems to be a normal result that cellular losses in apoptosis, which is programmed cell death, are associated with ulcerative lesions in histopathological findings, shortened villus lengths and decreased PCNA expressions, which are proliferation indicators in immunostaining. On the other hand, the levels of apoptotic BAX and antiapoptotic BCL2 gene expressions, whose gene expressions are examined by PCR method in genetic analyzes, can be used to support apoptosis. It is known that increases in the BAX gene and decreases in the BCL2 gene, which are located in the membrane of the mitochondria organelle, which is known to have important roles in respiration and energy at the cellular level, cause an increase in mitochondrial membrane permeability and lead to apoptosis with increased Cas-3 mediated by cytochrome c and caspase-9 26. Similar findings found in the MIR group in this study are another indication that intestinal cells go to apoptosis through mitochondrial damage. The limitation of this study is that cytochrome c and caspase 9 gene expressions, which are other genes between these mitochondrial membrane genes and Cas-3, were not examined in this study. Significant reversal of all these findings in Tr or Pr RF-EMF treated groups suggests that the methods may have direct antiapoptotic effect or may inhibit hypoxia secondary to inflammation.

Oxidative stress parameters, which are more gross biomarkers, result in an increase in oxidant load and a decrease in antioxidant substances in damage situations. Antioxidant substances, whose synthesis or activity increases, are consumed and their levels decrease in order to eliminate the increased oxidant substances that occur in the fight against damage 27. The non-significant changes in TOS and TAS indicators detected in this study are insufficient to determine the stage of the described conditions. However, looking at OSI values, which are TOS/TAS indicators, would be a more realistic approach to show oxidative stress. Increases in OSI values in the MIR groups of the study indicate that oxidative stress develops in the damage group and decreases in the treatment groups indicate that oxidative stress can regress. This may be interpreted as RF-EMF applications may show antioxidant activity by directly increasing antioxidant enzyme activity or prevent the formation of oxidant substances due to its proximity to the sham group and keep antioxidants high. In order to clarify this situation, it is necessary to study the groups in which RF-EMF was applied alone without the damage group. This is among the limiting factors of this study.

The experimental model for the development of the above-mentioned inflammation, oxidative stress and apoptosis-related damages is hypoxia. It is known that hypoxia triggers VEGF expression, which is an indicator of both angiogenesis and inflammation, through activation of HIF1α, an important intracellular pathway, and inflammation through inducible nitric oxide synthase (INOS) 28. The association of HIF1α and VEGF expression increases with histopathologic and immunohistochemical findings in the MIR groups explains this situation. The decrease in HIF1α and VEGF expressions in RF-EMF groups and the similarity with the sham group indicate that the HIF1α pathway may be suppressed. In fact, an increase is observed in eNOS gene expressions, which are activated by the same pathway and may provide an effect on tissue blood supply by increasing NO production, in case of inflammation. Looking at the eNOS data of this study, it was observed that RF-EMF applications increased eNOS expressions and this was especially significant in the Pr group. This shows that RF-EMF treatment causes an increase in expression in the intact vascular endothelium and protects the intestinal tissue, especially before the model is created, that is, before ischemia is induced. Therefore, it can be interpreted that HIF1α could not increase due to decreased hypoxic environment and prevented damage.

The limitations of our study are as follows: 1- The use of only female animals, 2- The inability to investigate detailed cellular signaling mechanisms, 3- The inability to assess the protein-level expressions of the examined genes due to financial constraints, 4- The lack of evaluation of RF-EMF effects over long periods and at varying doses. In our future studies, we aim to include animals of both sexes to determine whether the effects of RF-EMF are sex-dependent. Additionally, it is important to investigate various signaling mechanisms in detail and document their protein-level expressions to contribute to the scientific literature.

## Conclusions

RF-EMF-treatment preserved the vascularization of the tissue and decreased hypoxia-induced oxidative stress, inflammation and apoptosis. This situation needs to be supported by more comprehensive studies that can be applied without damage, the effect of NO on platelets in ischemic area, where protein level expressions such as western blot can also be examined and different pathways can be investigated.

## Figures and Tables

**Table 1:** Scores of histopathological findings

Criteria	0	1	2	3
**Hyperemia**	None	Slight	Moderate	Severe
**Hemorrhage**	None	Only 3-5 erythrocytes	Small hemorrhagic areas	Marked hemorrhage
**Edema**	None	Slight	Moderate	Severe
**Infiltration**	No infiltration	Mild inflammatory cell infiltrates	Moderate inflammatory cell infiltrates	Severe inflammatory cell infiltrates
**Erosion/Ulcers**	No lesion	Superficial erosions	Ulcers that do not reach the submucosa	Ulcers reach the submucosa

**Table 2:** Primary sequences, product size and accession numbers of genes

Genes	Primary sequence	product size	accession number
**GAPDH (Housekeeping)**	F: AGTGCCAGCCTCGTCTCATA	248 bp	NM_017008.4
	R: GATGGTGATGGGTTTCCCGT		
**VEGF**	F: TTCGTCCAACTTCTGGGCTC	482 bp	NM_001287111.1
	R: GCTTTCTGCTCCCCTTCTGT		
**BCL2**	F: CATCTCATGCCAAGGGGGAA	284 bp	NM_016993.2
	R: TATCCCACTCGTAGCCCCTC		
**BAX**	F. CACGTCTGCGGGGAGTCAC	419 bp	NM_017059.2
	R: TAGAAAAGGGCAACCACCCG		
**eNOS**	F: GGTTGACCAAGGCAAACCAC	247 bp	NM_021838.2
	R: CCTAATACCACAGCCGGAGG		
**HIF1 α**	F: GCAACTAGGAACCCGAACCA	251 bp	NM_024359.2
	R: TCGACGTTCGGAACTCATCC		

F: Forward, R: Reverse, GAPDH: Glyceraldehyde-3-phosphate dehydrogenase BAX: BCL2 Associated X, BCL2: B-cell lymphoma 2, HIF1 α: Hypoxia-inducible factor 1 alpha, VEGF: Vascular Endothelial Growth Factor, eNOS: Endothelial nitric oxide synthase
